# Producing Contour Fillings

**Published:** 1892-11

**Authors:** R. Ottolengui


ARTICLE II.

### PRODUCING CONTOUR FILLINGS.

BY R. OTTOLENGUI.

When attendant circumstances do not contra-indicate its use, gold undoubtedly will give the greatest satisfaction where any considerable portion of a tooth must be reproduced. I have known men who have claimed that they could make a permanent contour with *non*-cohesive gold. I do not doubt that such men do what they claim, but these individuals are rare. Therefore, I advise the beginner to depend on *cohesive* gold when his filling must extend beyond cavity-borders. Moreover, I would suggest that he obtain gold *as cohesive as it can be made.*

There are methods of manipulating gold, essential when contour is to be reproduced, which may not be so in ordinary cavities. For example, a filling might be made hollow, provided it touched the walls at all points. This would be a grand error in a contour filling. It has been stated by some that a large cavity may be well filled with crystal gold, the inner portion being only partly condensed, provided the outer third be made solid. However, this may be in ordinary work, it is not to be thought of in contouring. In placing a contour filling every piece of gold, from the first to the last, must be thoroughly condensed, and each pellet should display perfect cohesion. To take up these two points separately for a moment: Let us suppose an ordinary cavity with surrounding walls; it is half filled; the operator places a pellet, and mallets it less thoroughly than he has done its predecessors. He adds another, and continues the malleting: Of course, the force of the blows will further condense the under pellet. Even if it be not completely condensed, the fact that the shape of the remainder of the cavity is retentive without regard to that part already filled, makes it of no moment whether that one pellet is, or is not, thoroughly packed. With a contour filling it would be a hazardous oversight to leave a single pellet of gold insufficiently condensed. That point would be a weak spot. If heavy foil is being used, the rule is even more imperative. Every layer must be thoroughly condensed before the next is added, because with this kind of gold more than any other, an underlying layer is less likely to be condensed where another is superimposed before the malleting is completed. For a similar reason, however much need there may be to hurry, the dentist should never pick up two or three pieces of heavy foil at one time and attempt to condense them in that form; the ends, being irregularly arranged, will fold one over the other in such shapes as to offer the greatest possible resistance to the mallet, the result being improper condensation.

With the other point, relative to cohesion, the necessities for extreme caution, extending to every pellet, become evident along the same lines of argument. It is as bad for a filling to fracture because one layer did not cohere, as because there was a flaw from lack of solidity. As before, non-cohesion, or slight cohesion, may be overlooked in the body of a cavity having surrounding walls, because what is placed above it will still be retained by the upper part of the cavity. It is otherwise with the contour filling. If only one layer, especially if it be of heavy foil, fails to cohere, all that which follows is but added to, and is not a part of, the filling. A fracture may be expected at any time.

It, therefore, follows that the size of the pellets, or strips of heavy foil, should not be increased near the end of the filling to hurry the work. Larger pieces will render solidity and cohesion both less liable. Above all things, large or even moderate-sized foot pluggers are to be avoided, though more permissible with the Bonwill mechanical, or the electrical mallet, than where a hand-mallet or hand-pressure is relied on. I wish to condemn the foot-plugger for this class of work, yet must speak with caution. Many men of skill use the foot-plugger with success, and with more rapidity than where another form was chosen. But these men select a foot-plugger which is narrow, and reaches a sharp point at one end. Thus, in one instrument is had the action of a foot-plugger, or of a point. What I am advising against is a broad, flat, and unusually long foot-plugger. This condenses so much surface at once that thorough cohesion is doubtfully obtained.

One more essential point: In packing any filling I make it my rule that, from the very initial pieces, the shape of the cavity must be such that I can use the mallet, without needing another tool to hold the gold steady. This is the rule; exceptions exist, but are very rare. *Applied to contour fillings, the rule must have absolutely no exceptions.* Every piece of gold as it is added must produce a filling, as far as the work has progressed, to dislodge which would require the engine drill. If at any time it is found that the filling will tip, or move in the slightest, the operator may as well remove his gold, and reshape his cavity. To emphasize this point, I will relate a case which occurred early in my career.

I was asked by a dentist, whose experience and skill were much respected by myself, to be at his office one afternoon to assist him in placing a large contour filling. He wished me to mallet and pass the gold. I did so. The tooth was a central incisor. The operator had prepared a cavity retentive in shape, merely because with a wheel-bur he had made an undercut, or groove, all around. This is exactly the plan to be followed in similar cases, where from abrasion the ends of teeth have been worn away, and it is desired to stop the destruction by offering masticating surfaces of gold. Then the filling is made *flush with the top of the tooth*, and will remain in place even though it must be held with another instrument all through the process of packing. (This, of course, need not be if the groove be properly shaped to retain the first pellet.) To so arrange a cavity, however, where, as in the case which I am citing, *about one quarter of the length of the tooth was to be restored*, was absurd. Thus I thought, but of course made no comment. The dentist began with a rather large pellet, and proceeded much as though he had been using *non*-cohesive gold. That is, he was mainly depending on wedging the pieces across from one groove to the other, using one instrument for condensing under the force of the mallet wielded by myself, and another to prevent the gold from rocking or moving. This was kept up till the whole cavity was filled flush, when of course it *appeared* solid. Then the work progressed rapidly, till the whole contour was completed, at the end of two hours' work. *In finishing this filling with a file, the dentist succeeded in straining it from its anchorage so that it could be slightly rocked.* He looked at me silently, and I refrained from speaking. He tipped it out, and, strange to say, proceeded to refill the cavity without change of plan. This time he succeeded in making the contour, and also in polishing it so that it *looked* really handsome. Moreover, it lasted as long as the lady lived, though I should record that *she died two weeks later*.

The essential features of a gold contour, therefore, are extreme solidity, extreme cohesion, and extreme immobility throughout and at every stage of the operation.—*Cosmos*.

